# Polygenic Score Models for Alzheimer’s Disease: From Research to Clinical Applications

**DOI:** 10.3389/fnins.2021.650220

**Published:** 2021-03-29

**Authors:** Xiaopu Zhou, Yolanda Y. T. Li, Amy K. Y. Fu, Nancy Y. Ip

**Affiliations:** ^1^Division of Life Science, State Key Laboratory of Molecular Neuroscience and Molecular Neuroscience Center, The Hong Kong University of Science and Technology, Hong Kong, China; ^2^Hong Kong Center for Neurodegenerative Diseases, Hong Kong Science Park, Hong Kong, China; ^3^Guangdong Provincial Key Laboratory of Brain Science, Disease and Drug Development, HKUST Shenzhen Research Institute, Shenzhen–Hong Kong Institute of Brain Science, Shenzhen, China

**Keywords:** Alzheimer’s disease, polygenic score, *APOE*, genetics, polygenic risk score, polygenic hazard score, risk prediction

## Abstract

The high prevalence of Alzheimer’s disease (AD) among the elderly population and its lack of effective treatments make this disease a critical threat to human health. Recent epidemiological and genetics studies have revealed the polygenic nature of the disease, which is possibly explainable by a polygenic score model that considers multiple genetic risks. Here, we systemically review the rationale and methods used to construct polygenic score models for studying AD. We also discuss the associations of polygenic risk scores (PRSs) with clinical outcomes, brain imaging findings, and biochemical biomarkers from both the brain and peripheral system. Finally, we discuss the possibility of incorporating polygenic score models into research and clinical practice along with potential challenges.

## Introduction

Alzheimer’s disease (AD), an aging-related neurodegenerative disease and the most common form of dementia, is a health threat to societies worldwide. AD has a complex etiology that is influenced by both genetic and environmental factors, which account for its variable risk among individuals. The presence of known coding mutations located in *APP* and *PSEN* genes that exhibit extremely high disease penetrance for early-onset AD can be determined by genetic analysis well before disease onset. Moreover, sporadic late-onset AD (LOAD), which accounts for most AD cases, is suggested to be highly heritable (approximately 60–80%) in the general population (Gatz et al., 2006). Therefore, studying individual genomes might identify individuals at high risk of developing AD, create a time window for intervention, and aid the development of intervention strategies.

However, genome-wide association studies (GWASs) of LOAD have only revealed a few dozen genetic risk loci with mild or moderate disease risk-modifying effects; individually, these cannot adequately explain an individual’s risk of having AD at the population level (Lambert et al., 2013; Jansen et al., 2019). The inconsistencies among epidemiological studies regarding the high heritability of LOAD as well as the lack of causal genetic factors that adequately explain disease risk imply that LOAD has a polygenic nature: its risk might be modulated by the aggregate effects of many hidden variants as well as environmental factors. Accordingly, given that polygenic risk analysis has recently become a key facet in cohort studies of LOAD, herein we systemically review the current approaches to polygenic risk analysis along with their applications in AD.

## Key Elements of Polygenic Score Models

Polygenic score models consider the aggregate effects of multiple variants to evaluate genetic contributions to continuous or discrete traits—for instance, gene expression levels or disease status (Chatterjee et al., 2016). Hence, polygenic score models require knowledge about which variants modify the disease in question. Variants are normally selected by screening the summary statistics generated by GWASs with proper filtering of the association *p*-values. Various *p*-value thresholds can be applied (e.g., 0.0001, 0.01, or 0.5) to obtain the pools of variants that exhibit optimal performance for AD classification (Escott-Price et al., 2019b). Meanwhile, several methods have been applied to overcome the redundancy of genetic information (i.e., the effects of the variants on a given disease) due to high linkage disequilibrium among selected variants. For instance, linkage disequilibrium-based pruning, which removes variants in high linkage disequilibrium, or linkage disequilibrium-aware clumping, which simultaneously removes variants in high linkage disequilibrium while retaining variants with the smallest *p*-values, have been applied to select the most informative variants to construct a polygenic score model. In addition to *p*-value–based selection, other statistical learning methods such as lasso regression, which can select the most informative variants for AD classification by removing variants minimally associated with the disease, have been also incorporated into polygenic risk analysis for AD (Romero-Rosales et al., 2020; Zhou et al., 2020).

Once the variants for model construction have been determined, their genotype dosages are summarized into a single value that can represent an individual’s status (i.e., their relative risk of having AD). The easiest way to achieve this is to simply sum the number of risk alleles across all selected variants to generate an unweighted polygenic score (Tosto et al., 2017). Meanwhile, two types of weighting measures are commonly introduced into polygenic score models to account for the variable impacts of individual variants on disease risk and generate a more accurate polygenic score model. First, the effect size can be determined from an association test, meta-analysis, or log-transformed odds ratios, thus yielding a weighted polygenic risk score (PRS) model (Tosto et al., 2017). Second, log-transformed hazard ratios generated from association analysis for disease onset age can also be introduced to produce a polygenic hazard score (PHS), which indicates an individual’s instantaneous risk of developing a given disease (Tan et al., 2018).

Nevertheless, introducing statistical learning methods into polygenic risk analysis enables simultaneous variant selection and model construction. Such methods, including lasso regression and support vector machines, can directly learn from the raw genotype data and use the same framework to construct models to predict various outcomes (e.g., phenotypes, cognitive performance, and onset age). Moreover, they may perform better than PRS and PHS models given their ability to better capture both local and global genomic structures.

## Overview of Polygenic Score Research for Alzheimer’s Disease

**FIGURE 1:**
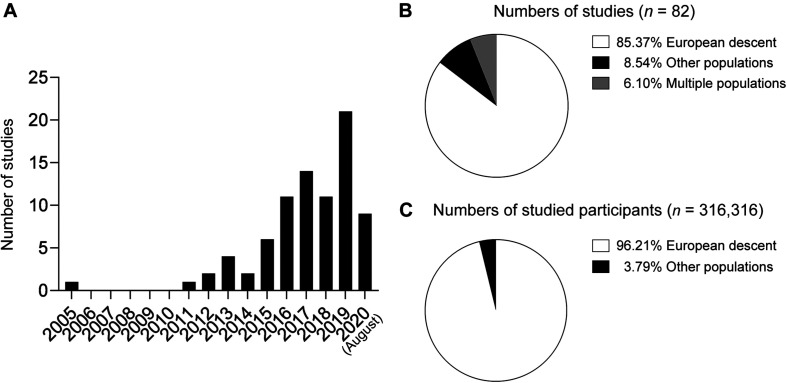
Summary of polygenic score research on Alzheimer’ disease. **(A)** Numbers of published papers by year. **(B)** Proportions of studies by population. **(C)** Proportions of study participants by ethnic group.

The number of published research articles associated with AD polygenic score models has dramatically increased over the last 15 years (Figure 1A). In 2005, one study reported an AD polygenic score model constructed from nine cholesterol-related single nucleotide polymorphisms (SNPs) including *APOE*-ϵ4 that exhibited superior performance for classifying AD compared to *APOE*-ϵ4 alone [area under the receiver operating characteristic curve (AUC) = 0.74 vs. 0.66 for the polygenic score model and *APOE*-ϵ4, respectively] (Papassotiropoulos et al., 2005). That study was also the first to demonstrate the applicability of polygenic score models to predict AD risk—even before AD GWASs demonstrated the polygenic nature of AD.

Large-scale AD GWASs in populations of European descent bolstered AD polygenic score research in recent years by providing comprehensive information about the effects of individual variants on AD risk at a genome-wide scale. Those studies’ summary statistics, which contain the effect sizes of individual variants, can be directly applied as weighting factors to construct a PRS model. In fact, several AD polygenic risk studies were based on the summary statistics generated by the IGAP Consortium published in 2013 (Lambert et al., 2013) and investigated AD polygenic score models in populations of European descent (Marden et al., 2014, 2016; Escott-Price et al., 2015, 2017a,b, 2019a,b; Habes et al., 2016; Harrison et al., 2016; Louwersheimer et al., 2016; Lupton et al., 2016; Mormino et al., 2016; Walter et al., 2016; Darst et al., 2017; Desikan et al., 2017; Foley et al., 2017; Gibson et al., 2017; Hayes et al., 2017, 2020; Marioni et al., 2017; Morgan et al., 2017; Tan et al., 2017, 2019; Xiao et al., 2017; Axelrud et al., 2018, 2019; Cruchaga et al., 2018; Del-Aguila et al., 2018; Ge et al., 2018; Kauppi et al., 2018, 2020; Patel et al., 2018; Stephan et al., 2018; Tasaki et al., 2018; Andrews et al., 2019; Chaudhury et al., 2019; Elman et al., 2019; Guerreiro et al., 2019; Korologou-Linden et al., 2019a,b; Kremen et al., 2019; Lancaster et al., 2019; Leonenko et al., 2019a,b; Logue et al., 2019; Wang et al., 2019; Yu et al., 2019; Ajnakina et al., 2020; Han et al., 2020; Matloff et al., 2020; Reus et al., 2020; Yesavage et al., 2020). Meanwhile, a few other studies focusing on populations of non-European descent also applied the IGAP data to select variants for genotyping analysis (Marden et al., 2014; Tosto et al., 2017; Axelrud et al., 2018, 2019; Li et al., 2020). Notably, the study populations of most AD polygenic risk studies (Figure 1B) and studied individuals (Figure 1C) were of European descent.

The availability of GWAS results from AD genetics studies has enabled the selection of variants for model construction. Studies using the same IGAP summary statistics can generate models with different numbers of variants (from 6 to 1.1 million sites) by selecting different *p*-value thresholds (Ajnakina et al., 2020; Han et al., 2020; Reus et al., 2020). Meanwhile, the sample sizes used for polygenic score models also vary among studies: from less than 80 to more than 20,000 participants (Desikan et al., 2017; Chandler et al., 2019). Regarding model construction, PLINK and PRSice are the most widely used tools to select variants and construct polygenic score models. Other statistical analysis methods, such as linear support vector machine (Filipovych et al., 2012), lasso regression (Romero-Rosales et al., 2020; Zhou et al., 2020), multilocus genotype patterns analysis (Barral et al., 2012), and decision tree (Yokoyama et al., 2015; Porter et al., 2018c), have also been adopted to construct polygenic score models for AD.

Of note, polygenic score models have been implemented to investigate the effects of genetic variants on various aspects of AD pathogenesis and progression. Most studies focus on clinical outcomes, specifically the classification of patients with AD (Papassotiropoulos et al., 2005; Sabuncu et al., 2012; Marden et al., 2014; Adams et al., 2015; Escott-Price et al., 2015, 2017b, 2019b; Yokoyama et al., 2015; Lupton et al., 2016; Tosto et al., 2017; Xiao et al., 2017; Cruchaga et al., 2018; Patel et al., 2018; Chaudhury et al., 2019; Leonenko et al., 2019a,b; Zhang et al., 2019; Altmann et al., 2020; Andrews et al., 2020; Zhou et al., 2020). Some other studies investigated the possible associations between AD polygenic score models and the risk of conversion to AD or mild cognitive impairment (MCI) (Filipovych et al., 2012; Rodríguez-Rodríguez et al., 2013; Verhaaren et al., 2013; Carrasquillo et al., 2015; Desikan et al., 2017; Tosto et al., 2017; Kauppi et al., 2018; Chaudhury et al., 2019; Elman et al., 2019; Ajnakina et al., 2020; Altmann et al., 2020; Andrews et al., 2020), cognitive function (Louwersheimer et al., 2016; Del-Aguila et al., 2018; Ge et al., 2018; Kauppi et al., 2018, 2020; Porter et al., 2018a,c,b; Stephan et al., 2018; Tan et al., 2018, 2019; Tasaki et al., 2018; Korologou-Linden et al., 2019a; Han et al., 2020; Zhou et al., 2020), and memory function (Barral et al., 2012; Verhaaren et al., 2013; Marden et al., 2014, 2016; Adams et al., 2015; Carrasquillo et al., 2015; Mormino et al., 2016; Hayes et al., 2017; Marioni et al., 2017; Axelrud et al., 2018; Ge et al., 2018; Porter et al., 2018a,b,c; Tan et al., 2018, 2019; Altmann et al., 2020). Notably, given that the brain’s structure and functions are closely associated with cognitive ability, several studies have also investigated the use of polygenic score models to predict brain status including changes in brain structure (Sabuncu et al., 2012; Habes et al., 2016; Harrison et al., 2016; Nho et al., 2016; Desikan et al., 2017; Foley et al., 2017; Hayes et al., 2017, 2020; Xiao et al., 2017; Ge et al., 2018; Kauppi et al., 2018; Li et al., 2018; Tasaki et al., 2018; Chandler et al., 2019, 2020; Tan et al., 2019; Wang et al., 2019; Altmann et al., 2020; Matloff et al., 2020; Zhou et al., 2020) and function (Xiao et al., 2017; Axelrud et al., 2019; Chandler et al., 2019, 2020). Moreover, some studies investigated biochemical changes indicative of brain status, such as AD pathological hallmarks including amyloid-beta (Aβ) load and tau tangles (Mormino et al., 2016; Darst et al., 2017; Desikan et al., 2017; Laiterä et al., 2017; Ge et al., 2018; Porter et al., 2018a,b,c; Tan et al., 2018, 2019; Tasaki et al., 2018; Leonenko et al., 2019a; Yu et al., 2019; Altmann et al., 2020), enzyme activity in brain samples (Martiskainen et al., 2015; Laiterä et al., 2017), and levels of proteins (e.g., the “ATN” biomarker panel, which comprises Aβ, tau, and neurofilament light polypeptide) or metabolites (Papassotiropoulos et al., 2005; Sabuncu et al., 2012; Martiskainen et al., 2015; Louwersheimer et al., 2016; Mormino et al., 2016; Darst et al., 2017; Morgan et al., 2017; Cruchaga et al., 2018; Porter et al., 2018a; Tasaki et al., 2018; Korologou-Linden et al., 2019b; Tan et al., 2019; Altmann et al., 2020; Hayes et al., 2020; Li et al., 2020; Reus et al., 2020; Zhou et al., 2020). Some studies also used polygenic score models to evaluate the extent to which certain diseases or pathways modulate AD risk (Papassotiropoulos et al., 2005; Moskvina et al., 2013; Mukherjee et al., 2015; Walter et al., 2016; Gibson et al., 2017; Hayes et al., 2017; Demichele-Sweet et al., 2018; Creese et al., 2019; Elman et al., 2019; Guerreiro et al., 2019; Kremen et al., 2019; Lancaster et al., 2019; Andrews et al., 2020; Yesavage et al., 2020). Collectively, those studies suggest that genetic factors have crucial roles in modifying AD risk and highlight the potential utility of polygenic score models in AD research and routine clinical practice. In the following section, we summarize the key findings of each of those aspects.

## Polygenic Score Models for Predicting Alzheimer’s Disease Risk

The primary goal of a polygenic score model is to classify individuals according to disease risk (AD in this case). Numerous studies conducted in recent decades have established various polygenic score models and report their ability to adequately distinguish patients with AD from cognitively normal individuals. Reported AD prediction accuracy ranges from an AUC of 0.57 (Tosto et al., 2017) to 0.84 (Escott-Price et al., 2017a). Notably, Yokoyama et al. (2015) generated a PRS using a decision tree model and report an AUC of 0.88 for the prediction of AD (vs. 0.69 for *APOE* genotype) in their discovery cohort (*n* = 192). However, this model failed to surpass the accuracy of using *APOE* genotype to predict AD in their replication cohort (AUC = 0.62 vs. 0.63 for the PRS and *APOE* genotype, respectively; *n* = 276). In contrast, several other studies demonstrate that PRS models exhibit superior performance to *APOE* genotype for predicting AD or associated cognitive states as indicated by significant associations between AD and PRSs that do not include *APOE* genotype (Sabuncu et al., 2012; Xiao et al., 2015; Leonenko et al., 2019a,b; Zhang et al., 2019) or PRS results after controlling for *APOE* genotype (Tosto et al., 2017; Escott-Price et al., 2019b). Specifically, in one study recently published by Escott-Price et al. (2019b), the application of a PRS to homozygous *APOE*-ϵ3 carriers achieved an AUC of 0.831 for the prediction of AD with a comparable AUC of 0.834 after excluding the variants in the *APOE* region in homozygous *APOE*-ϵ3 carriers. Thus, polygenic effects might account for the non-*APOE*–dependent genetic mechanisms of AD pathogenesis. Meanwhile, a whole-exome sequencing study conducted by Patel et al. (2018) revealed the applicability of polygenic score models using exonic variants to predict AD, yielding an AUC of 0.830 for AD prediction with the inclusion of *APOE* genotype, age, sex, and 19 GWAS-identified SNPs, further implying the polygenic contribution of the exonic regions to the modulation of AD risk.

In addition to disease risk, a few studies investigated the possible contribution of polygenic risk to the modulation of the likelihood of AD conversion, specifically conversion from MCI to AD (Rodríguez-Rodríguez et al., 2013; Tan et al., 2017; Kauppi et al., 2018; Chaudhury et al., 2019) or conversion from cognitive normality to MCI or AD (Carrasquillo et al., 2015; Tan et al., 2017; Logue et al., 2019; Altmann et al., 2020), or the time to develop AD (Verhaaren et al., 2013; Desikan et al., 2017; Tosto et al., 2017; Ajnakina et al., 2020; Andrews et al., 2020). Of note, Tan et al. (2017) studied 1,081 asymptomatic elderly adults and report a PHS model based on 31 SNPs selected from IGAP and ADGC phase 1 data that can accurately predict the risk of conversion from cognitive normality to AD (hazard ratio = 2.36), from MCI to AD (hazard ratio = 1.17), and from cognitive normality or MCI to AD (hazard ratio = 1.31). Furthermore, Kauppi et al. (2018) integrated the PHS with cognitive score and brain atrophy status, resulting in relatively high accuracy for predicting conversion from MCI to AD (AUC = 0.84).

Notably, Carrasquillo et al. (2015) suggest that only *APOE*-inclusive PRSs are correlated with the likelihood of developing MCI or AD in a longitudinally assessed cohort. Moreover, Rodríguez-Rodríguez et al. (2013) also report that conversion from MCI to AD cannot be successfully predicted by PRSs after controlling for age, sex, and *APOE* genotype. However, the models in both studies included fewer than 10 non-*APOE* variants. Meanwhile, by integrating more variants into the analysis, Altmann et al. (2020) observed significant associations between AD polygenic risk and clinical conversion from non-demented to demented status as well as Clinical Dementia Rating Scale Sum of Boxes (CDR-SB) score after excluding the effect of the *APOE* locus. Therefore, the polygenic risk effects from non-*APOE* loci probably contribute to the likelihood of AD development and progression.

## Polygenic Score Models for Predicting Memory and Cognitive Functions

Besides disease states, polygenic risk is also correlated with individual memory function (Barral et al., 2012; Verhaaren et al., 2013; Marden et al., 2014, 2016; Adams et al., 2015; Carrasquillo et al., 2015; Mormino et al., 2016; Hayes et al., 2017; Marioni et al., 2017; Axelrud et al., 2018; Ge et al., 2018; Porter et al., 2018a,b,c; Tan et al., 2018, 2019; Altmann et al., 2020). Specifically, a multilocus mapping analysis conducted by Barral et al. (2012) demonstrates an association between episodic memory and specific genetic patterns from GWAS-identified variants; a few other studies also suggest possible associations between polygenic risk and episodic memory function. Specifically, a PRS study conducted by Marden et al. (2014) suggests that AD polygenic risk might modulate both baseline memory and its rate of decline in people of non-Hispanic European descent (*n* = 7,172) or African descent (*n* = 1,081). Again, there is some controversy about the effects of non-*APOE* polygenic risks on memory function. For instance, Carrasquillo et al. (2015) suggests that only *APOE*-inclusive PRSs are correlated with worsening memory function, while Verhaaren et al. (2013) and Porter et al. (2018b) report a significant association between non-*APOE* polygenic risk and memory function. Moreover, Ge et al. (2018) report a significant correlation between high AD polygenic risk and the rate of memory decline after controlling for *APOE*-ϵ4 genotype. Hence, the polygenic risk effects from non-*APOE* loci likely also influence memory function.

Polygenic scores can also indicate individual cognitive functions. Several studies report associations between polygenic risk and cognitive functions (Louwersheimer et al., 2016; Del-Aguila et al., 2018; Ge et al., 2018; Kauppi et al., 2018, 2020; Porter et al., 2018a,b,c; Stephan et al., 2018; Tan et al., 2018, 2019; Tasaki et al., 2018; Korologou-Linden et al., 2019a; Han et al., 2020; Zhou et al., 2020). For instance, Korologou-Linden et al. (2019a) report an association between PRS and lower total, verbal, and performance intelligence quotients in childhood and adolescence, and Kauppi et al. (2020) suggest that AD polygenic risk is indicative of individual differences in the rate of cognitive decline in normal aging. Meanwhile, Xiao et al. (2017) and Li et al. (2018) did not identify a significant association between AD polygenic risk and cognitive function in cognitively normal individuals.

## Polygenic Score Models for Predicting Brain Status

The associations of polygenic scores with memory and cognitive function imply possible alterations of brain structure and functions. Several studies have examined the associations between AD polygenic risk and MRI findings (Sabuncu et al., 2012; Habes et al., 2016; Harrison et al., 2016; Mormino et al., 2016; Nho et al., 2016; Desikan et al., 2017; Foley et al., 2017; Hayes et al., 2017, 2020; Xiao et al., 2017; Ge et al., 2018; Kauppi et al., 2018; Li et al., 2018; Chandler et al., 2019; Tan et al., 2019; Wang et al., 2019; Altmann et al., 2020; Matloff et al., 2020; Zhou et al., 2020), fMRI findings (Xiao et al., 2017; Axelrud et al., 2019; Chandler et al., 2020), and PET imaging findings (Mormino et al., 2016; Darst et al., 2017; Ge et al., 2018; Porter et al., 2018a,b,c; Tan et al., 2018, 2019; Leonenko et al., 2019a; Altmann et al., 2020).

Notably, some studies have examined the associations between AD polygenic risk and the volumetric changes of various brain regions such as the retrosplenial and posterior cingulate cortices (Sabuncu et al., 2012), frontal cortex (Chandler et al., 2019), entorhinal cortex (Harrison et al., 2016; Desikan et al., 2017; Hayes et al., 2020), amygdala (Lupton et al., 2016; Zhou et al., 2020), and hippocampus (Harrison et al., 2016; Lupton et al., 2016; Desikan et al., 2017; Foley et al., 2017; Xiao et al., 2017; Axelrud et al., 2018; Ge et al., 2018; Lancaster et al., 2019; Altmann et al., 2020; Hayes et al., 2020; Matloff et al., 2020; Zhou et al., 2020).

Interestingly, some studies have focused on individuals of varying ages including young adolescents (Li et al., 2018; Chandler et al., 2019) and elderly people (Lupton et al., 2016; Nho et al., 2016; Darst et al., 2017; Desikan et al., 2017; Tan et al., 2019; Hayes et al., 2020). Specifically, Li et al. (2018) and Chandler et al. (2019) report significant associations of AD polygenic risk with gray matter cerebral blood flow and gray matter volume, respectively, in young individuals, indicating a potential long-term effect of polygenic risk on brain function well before AD onset.

In addition to structural changes, AD polygenic risk might be associated with brain Aβ load (Mormino et al., 2016; Darst et al., 2017; Porter et al., 2018a,c; Tan et al., 2018, 2019; Leonenko et al., 2019a; Altmann et al., 2020) as measured by PET imaging. Moreover, several studies discuss the possible effects of polygenic risk on brain functional changes including hippocampal activation (Xiao et al., 2017; Chandler et al., 2020) and connectivity between specific brain regions (Axelrud et al., 2019), providing additional evidence for the effects of AD polygenic risk on brain function. Meanwhile, Aβ measured by PET imaging has been introduced to stratify AD patients prior to PRS evaluation (Porter et al., 2018b).

## Polygenic Score Models for Predicting Biochemical Changes in the Brain and Peripheral System

Corroborating PET imaging findings, AD polygenic risk is also associated with the levels of several hallmark proteins of AD in postmortem brain tissues. For instance, AD PRSs are reported to be significantly correlated with Aβ and tau tangle levels (Tasaki et al., 2018), although some studies did not identify such a correlation between AD polygenic risk and Aβ levels (Laiterä et al., 2017; Yu et al., 2019). Notably, AD polygenic risk might be correlated with the activity of brain γ-secretase (but not β-secretase) (Martiskainen et al., 2015; Laiterä et al., 2017) as well as levels of VGF, IGFBP5, and STX1A in brain tissues as measured by proteomic analysis (Tasaki et al., 2018).

As the ATN biomarkers in cerebrospinal fluid (CSF) are correlated with the brain pathology in AD, several studies also suggest possible correlations between PRSs and CSF biomarkers including Aβ (Sabuncu et al., 2012; Martiskainen et al., 2015; Darst et al., 2017; Cruchaga et al., 2018; Hayes et al., 2020; Li et al., 2020) and tau or p-tau (Louwersheimer et al., 2016; Darst et al., 2017; Cruchaga et al., 2018; Porter et al., 2018a; Tan et al., 2018; Altmann et al., 2020; Li et al., 2020; Reus et al., 2020). However, Louwersheimer et al. (2016) and Mormino et al. (2016) did not observe a correlation between AD polygenic risk and CSF Aβ levels. Meanwhile, Reus et al. (2020) examined the associations between polygenic risk and 412 CSF proteins and protein fragments, and found that 48.8% of the candidate proteins were associated with at least one of the 14 constructed scores, implying a possible global alteration of the CSF proteome that is possibly associated with polygenic risk.

Notably, a recent study also implies the involvement of the peripheral immune system in AD pathogenesis (Zhou et al., 2018), while other studies demonstrate associations between AD polygenic risk and plasma proteins (Morgan et al., 2017; Korologou-Linden et al., 2019b; Zhou et al., 2020) or metabolites (Papassotiropoulos et al., 2005; Korologou-Linden et al., 2019b). Specifically, by applying the proximity extension assay to plasma proteomic analysis, we investigated 280 proteins and revealed potential protein candidates (i.e., osteopontin and neurocan core protein) along with a protein network associated with AD polygenic risk—again implying global changes in plasma profiles that might be modulated by polygenic risk (Zhou et al., 2020).

## Polygenic Score Models for Examining the Involvement of Other Diseases in Alzheimer’s Disease Pathogenesis

The complex etiology of AD is reflected by the identification of various modifiable risk factors such as cardiovascular risk factors, hypertension, and immune factors. Polygenic score models suggest that AD genetic risks are associated with cholesterol levels (Papassotiropoulos et al., 2005), depression (Gibson et al., 2017), schizophrenia (Demichele-Sweet et al., 2018; Creese et al., 2019), frontotemporal lobar degeneration, amyotrophic lateral sclerosis (Adams et al., 2015), insulin sensitivity (Walter et al., 2016), microglial dysfunction (Lancaster et al., 2019), and mitochondrial dysfunction (Andrews et al., 2020). Meanwhile, the polygenic risks for cardiovascular risk factors, frontotemporal lobar degeneration, and amyotrophic lateral sclerosis are implicated in the pathogenesis of MCI (Adams et al., 2015; Elman et al., 2019). Thus, these findings collectively suggest the underlying mechanisms of AD comorbidities and indicate possible pathways for intervention.

## Applications and Potential Issues

**FIGURE 2:**
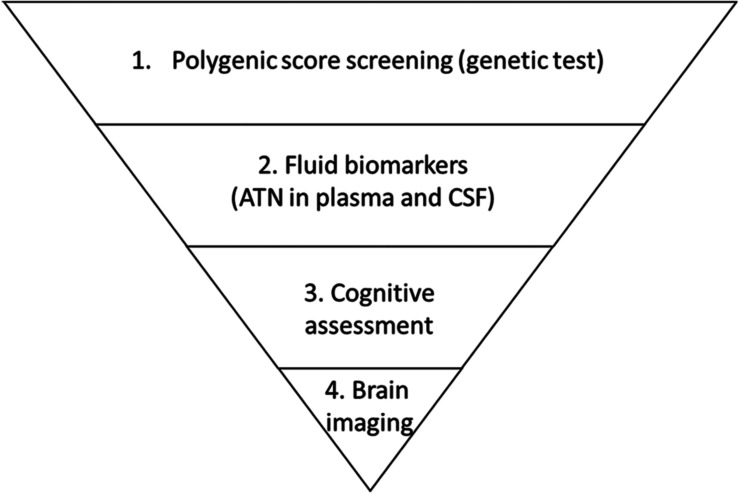
Proposed hierarchal strategy for Alzheimer’s disease risk screening. Individuals enrolled in a screening task are first examined according to genetic risk as indicated by polygenic risk analysis. Individuals who have relatively high risk and report symptoms are referred for biomarker examination to evaluate amyloid-beta, tau (and p-tau), and neurofilament light polypeptide levels (i.e., the “ATN” panel) in blood or cerebrospinal fluid (CSF). Those who exhibit altered levels of biomarkers are further referred to clinicians for cognitive assessment followed by brain imaging including magnetic resonance imaging and positron emission tomography.

Given the high prevalence of AD, early risk prediction might facilitate early intervention and greatly mitigate the future growth of the AD patient population. Specifically, polygenic risk factors rooted in individual genomes can be used as biomarkers for the early assessment of relative risk at a population scale. To illustrate the utility of such a strategy, delaying disease onset by 5 years would reduce the predicted AD population among people aged 70 years or above by 41% in the United States. in 2050 (Zissimopoulos et al., 2015). Furthermore, inspiring work by Solomon et al. (2018) further suggests that lifestyle interventions might override the risk effects of *APOE*-ϵ4, implying a possible means of delaying AD onset once an individual is informed of their relative risk of developing AD. Moreover, a recent study revealed that prior knowledge of genetic risk would also be critical for drug discovery, as drugs targeting proteins encoded in genetic risk loci would be more likely to be successful in phase II and III clinical trials (King et al., 2019). Notably, a polygenic score study of coronary heart disease risk showed that compared to people with lower genetic risk, those with higher genetic risk exhibited a greater decrease in absolute disease risk after receiving statin therapy (Mega et al., 2015). Therefore, conducting population-scale genetic screening for AD might simultaneously support the development of intervention strategies and enable the stratification of individuals according to their risk of AD based on their genetic patterns. More specifically, a hierarchal screening strategy for AD risk evaluation combining genetic, circulatory factors, and brain imaging techniques can be implemented at a populational scale to facilitate disease risk screening and clinical research on personalized interventions in a genotype-aware manner (Figure 2).

Nevertheless, there are potential issues that could hinder the development and implementation of polygenic scoring in routine clinical practice. First, policies protecting patient privacy must be carefully considered, because the results of one person’s genetic test might not only indicate their own risks of certain diseases but also those of their close relatives (Clayton et al., 2019). Second, the possible consequences of informing certain individuals about their estimated genetic risks for certain diseases must be carefully considered, as this could have positive and/or negative outcomes. Fortunately, after receiving brain amyloid imaging, cognitively normal people with elevated amyloid loads tend to make more changes to their lifestyle and future plans than those who do not have elevated amyloid loads (Largent et al., 2020). In addition, in one recent study, providing genetic test results illustrating the 3-year risk of developing AD to patients with MCI did not increase the risk of anxiety or depression (Christensen et al., 2020). Meanwhile, different diagnostic criteria across study cohorts might introduce bias into genetics studies and the subsequent construction of polygenic score models, although this can be reduced or eliminated by further incorporating other biomarkers to refine clinical diagnosis (Escott-Price et al., 2017a). Furthermore, the application of polygenic score models can help refine the results of genetic analyses based on control cohorts (i.e., controls in whom the disease of interest has not been investigated in detail) by ruling out individuals at risk of developing diseases (Escott-Price et al., 2019a). Moreover, polygenic score models may be used to define an individual’s risk of having a specific neurodegenerative disease, as studies have demonstrated that such models (or the genotyping of specific variants) can predict the risk of Parkinson’s disease (Nalls et al., 2016), Huntington’s disease (Kremer et al., 1994), amyotrophic lateral sclerosis (Saez-Atienzar et al., 2021), and multiple sclerosis (The International Multiple Sclerosis Genetics Consortium (IMSGC), 2010). In addition, polygenic score models may help estimate the effects of aging on disease risk. Finally, conducting polygenic risk analysis requires the availability of population-specific genetic risk information at the single-variant level. We previously showed that a polygenic score model based on the Chinese population performs poorly when applied to an AD cohort of European descent (Zhou et al., 2020). The poor performance of that polygenic score model can be explained by the differences in the genomic structures between populations of East-Asian and European descent. Given that there are limited AD GWASs on populations of non-European descent (Zhou et al., 2018; Kunkle et al., 2020), it is critical to comprehensively analyze AD genetic risk in such populations to facilitate the development of polygenic score models and their associated applications in populations worldwide.

## Author Contributions

XZ, AF, and NI outlined and wrote the review. XZ and YL conducted the literature review and data organization. All authors contributed to the article and approved the submitted version.

## Conflict of Interest

The authors declare that the research was conducted in the absence of any commercial or financial relationships that could be construed as a potential conflict of interest. The handling editor declared a past collaboration with one of the authors NI.
